# Artificial Intelligence Centrality in Psychotic Delusions and Violence Risk in Forensic Psychiatry: Retrospective Observational Study of Judicial Decisions

**DOI:** 10.2196/93349

**Published:** 2026-05-20

**Authors:** Alexandre Hudon, Chanel Pagé

**Affiliations:** 1 Department of Psychiatry and Addictology Faculty of Medicine Université de Montréal Montréal, QC Canada; 2 Forensic Psychiatry Unit Department of Psychiatry Institut universitaire en santé mentale de Montréal Montreal, QC Canada; 3 Centre de recherche de l'Institut universitaire en santé mentale de Montréal Montreal, QC Canada; 4 Department of Psychiatry Institut national de psychiatrie légale Philippe-Pinel Montreal, QC Canada; 5 Centre de pédagogie en sciences de la santé Université de Montreal Montreal, QC Canada

**Keywords:** artificial intelligence, AI, psychotic disorders, delusions, violence risk, forensic psychiatry, risk assessment, schizophrenia, insight, treatment adherence, dangerousness

## Abstract

**Background:**

Artificial intelligence (AI)–themed delusions are increasingly observed in psychotic-spectrum disorders, reflecting the incorporation of contemporary sociotechnical elements into delusional systems. However, it remains unclear whether the structural role of AI within these belief systems is associated with increased violence risk or more restrictive forensic outcomes. Given the importance of dynamic clinical factors (eg, insight and treatment adherence) in forensic risk assessment, clarifying the role of AI centrality has clinical and legal relevance.

**Objective:**

This study examined whether AI centrality within psychotic delusional systems is associated with (1) violence toward others and (2) judicial findings of significant public safety risk in forensic psychiatric decisions.

**Methods:**

This retrospective observational study used jurisprudential data from the Société québécoise d’information juridique database, including all publicly available Quebec tribunal and court decisions up to December 31, 2025. Eligible cases (N=29) involved psychotic-spectrum disorders with explicit AI-related delusional content and judicial consideration of dangerousness or disposition. The unit of analysis was the judicial decision. AI centrality was coded as central (n=15, 51.7%) or noncentral (n=14, 48.3%) using a structured, text-based framework. The primary outcome was documented violence toward others; secondary outcomes included direct AI-linked violence attribution and judicial findings of significant public safety risk. Covariates included impaired insight, treatment nonadherence, substance use disorder, and prior violence history. Data were extracted through full-text review using a standardized coding grid. Bivariate associations were analyzed using Fisher exact tests (α=.05), and odds ratios (ORs) with 95% CIs were calculated. Exploratory logistic regression models were performed to assess adjusted associations.

**Results:**

Violence toward others was documented in 20/29 (69%) cases. AI centrality was not significantly associated with violence (12/15, 80.0%, vs 8/14, 57.1%; OR 2.91, 95% CI 0.63-13.45; P=.26) but was strongly associated with direct AI-linked violence attribution (9/15, 60.0%, vs 2/14, 14.3%; OR 9.00, 95% CI 1.48-54.6; P=.01). Judicial findings of significant public safety risk were more frequent in AI-central cases (13/15, 86.7%, vs 9/14, 64.3%; OR 3.60, 95% CI 0.63-20.5; P=.24), although not statistically significant. AI-central cases demonstrated higher prevalence of impaired insight (13/15, 86.7%, vs 8/14, 57.1%; OR 4.89, 95% CI 0.79-30.1) and treatment nonadherence (9/15, 60.0%, vs 4/14, 28.6%; OR 3.75, 95% CI 0.74-18.9).

**Conclusions:**

AI centrality within delusional systems appears to be not independently associated with increased violence toward others but is strongly associated with AI-based attribution of behavior and markers of epistemic vulnerability, including impaired insight and treatment nonadherence. The findings suggest that AI-themed delusions function as structural organizers of meaning and agency rather than novel criminogenic risk factors. Clinically and legally, this underscores the importance of prioritizing dynamic risk variables over thematic novelty, informing more proportionate forensic decision-making and risk assessment in an era of rapidly evolving digital environments.

## Introduction

### AI‑Themed Delusions and Contemporary Techno‑Paranoia

Delusional content is not static; it reliably absorbs salient features of the sociotechnical environment, so “modern” threat agents (networks, devices, platforms) increasingly populate persecutory and passivity narratives. Evidence from a contemporary psychosis cohort suggests that technology‑related delusions are now common and may be rising over time, underscoring the clinical need to ask explicitly about internet and device-mediated beliefs during assessment and treatment planning [1]. Parallel work in nonclinical populations supports a continuum model in which technology‑focused fears range from realistic privacy concerns to implausible, self-referential, and frankly paranoid interpretations (captured empirically by the construct of “cyber‑paranoia”) [2]. In addition, systematic review evidence on social media and psychosis-spectrum “social brain” disorders synthesizes case-based and observational findings suggesting that algorithmic curation, ambiguity of online cues, and perceived surveillance can intensify suspiciousness and “ideas of reference” in vulnerable individuals, potentially strengthening delusional conviction through self-reinforcing digital “evidence” [3]. Against this backdrop, generative artificial intelligence (AI) introduces a qualitatively different affordance: continuous, interactive dialogue that can mirror affect, provide confirmatory narratives, and blur boundaries between inner experience and external “agentic” communication. Conceptual analyses have framed “AI psychosis” not as a new diagnosis but as a heuristic to study how prolonged human-AI interaction might trigger, reshape, or entrench delusional systems in predisposed users [4]. Recent open access commentary further argues that the principle (incorporating media or technology into delusions) is not new, but interactivity and “sycophantic” agreement may alter the risk profile by colluding with delusional elaboration rather than supporting reality testing [5,6]. These developments motivate forensic inquiry: when AI becomes the perceived persecutor, controller, or corroborating interlocutor, does tribunals’ reasoning about risk and disposition shift accordingly?

### Forensic Risk Factors, Insight, and Treatment Adherence in Psychosis

Forensic psychiatry distinguishes the presence of psychosis from the conditions under which risk escalates, emphasizing dynamic (modifiable) factors, such as active positive symptoms, substance use, insight, and adherence. Meta-analytic evidence indicates that schizophrenia and related psychoses are associated with elevated violence risk, but much of the excess risk appears mediated by comorbid substance use disorders. This highlights the importance of integrated substance use assessment and treatment in risk reduction [7]. Complementing this, a large systematic review and meta-regression [8[ of violence risk factors in psychosis identified robust associations for domains that include criminal history and several dynamic variables (eg, recent substance misuse and medication nonadherence), supporting the clinical primacy of treatable targets rather than diagnosis alone. Insight, in turn, is tightly linked to adherence and outcomes. In a large European observational study [8], better insight correlated with higher medication adherence and stronger therapeutic alliance, and modeled pathways indicated that insight can shape clinical severity indirectly via adherence and alliance. Medication nonadherence has also been associated with violence outcomes in large cohorts; for example, a community-based study [9] of patients with schizophrenia found nonadherence associated with higher odds of multiple categories of violence to others, even after rigorous confounder control strategies. In forensic settings, these relationships are clinically consequential because tribunals often evaluate not only past behavior but also the likelihood of relapse, destabilization, and risk escalation (processes in which insight and adherence are central). Accordingly, an AI‑themed delusion may be best conceptualized not as an unusual narrative but as a potential marker of delusional system organization and rigidity, which could correlate with impaired insight, refusal of treatment, substance misuse, and broader criminogenic histories.

### Legal Responses and Judicial Risk Assessment When Delusional Content Is Novel

Legal systems tasked with managing individuals found not criminally responsible on account of mental disorder (NCRMD) or insanity‑acquitted populations typically prioritize public safety, while balancing therapeutic needs and proportional restrictions. Canadian data illustrate that the NCRMD status is not a loophole. In a large comparative analysis [10], individuals found NCRMD were substantially more likely to be detained and remained under supervision longer than convicted counterparts, reflecting a risk management orientation rather than punitive sentencing logic. Within Quebec’s review board context, open access empirical work describing the *Commission d’Examen des Troubles Mentaux* (CETM) or *Tribunal Administratif du Quebec* (TAQ) practices emphasizes that dispositions (detention, conditional discharge, absolute discharge) are ordered in whether the person constitutes a significant threat to public safety and that tribunals rely heavily on treating teams’ clinical reports and structured approaches to risk formulation, with specific attention to standardized tools, such as the Historical Clinical Risk Management-20 (HCR‑20) [11]. At the same time, forensic guidance cautions against instrumental overreach and narrative bias: risk tools and risk formulations can become rhetorically compelling in ways that outstrip predictive precision, potentially fostering risk‑averse interpretations when cases contain vivid, emotionally salient material [12]. Systematic review evidence on structured professional judgment (SPJ) frameworks shows that SPJ-informed guidance (including HCR‑20‑based approaches) is increasingly used to support high-stakes secure care decisions but should function as an adjunct rather than a substitute for comprehensive case formulation and contextual clinical judgment [13]. AI‑themed delusions place pressure on this ecosystem of judgment because they can sound simultaneously plausible (given real surveillance or algorithmic personalization) and bizarre (given perceived sentience, omniscience, or mind control), heightening the interpretive ambiguity that courts and clinicians must manage. This makes judicial texts a valuable empirical window into how “novel” delusional content is translated (explicitly or implicitly) into risk rationales and legal dispositions.

### Objectives and Hypotheses

This study attempted to answer the following question: *How does the centrality of AI within psychotic delusional systems relate to forensic risk and judicial decision-making?* We sought to clarify whether AI-themed delusions function as contemporary narrative content or whether they occupy a structurally central role that meaningfully shapes behavior, risk trajectories, and review board dispositions. To meet this goal, the following specific objectives were defined:

- To systematically identify and characterize judgments in which AI is explicitly referenced within delusional content
- To classify the degree of AI centrality within the delusional system (central, marginal, or secondary) using a structured coding framework
- To examine the association between AI centrality and core forensic risk variables, including violence toward others, treatment refusal, lack of insight, substance use, and prior criminal history
- To analyze whether AI centrality is associated with judicial outcomes, including detention, conditional discharge, or unconditional discharge, as well as findings of significant public safety risk

Based on the available data in the current literature on delusional system organization, insight deficits, and violence risk in psychotic disorders, several hypotheses can be formulated:

- Cases in which AI occupies a central position within the delusional architecture could be associated with higher rates of impaired insight and treatment nonadherence, reflecting a more systematized and behaviorally organizing belief structure.
- AI centrality could be associated with an increased likelihood of violence toward others when the AI is construed as a persecutory or controlling agent that issues commands or justifies defensive or retaliatory actions.
- AI-central cases may demonstrate a higher probability of judicial findings of significant public safety risk and more restrictive dispositions compared to cases in which AI content is marginal or incidental.
- AI centrality will cluster with established criminogenic and clinical risk factors, suggesting that AI-themed delusions do not constitute a fundamentally new risk category but rather represent a contemporary technological expression of structurally organized psychosis within existing forensic risk frameworks.

## Methods

### Study Design and Data Source

This study followed a retrospective observational design using jurisprudential data extracted from publicly available legal decisions. The data source was the Société québécoise d’information juridique (SOQUIJ) database, which archives decisions from Quebec courts and administrative tribunals [14]. The study period extended from database inception to December 31, 2025. The study adhered to the RECORD (Reporting of Studies Conducted Using Observational Routinely Collected Health Data) guidelines and was further aligned with American Psychological Association (APA) Journal Article Reporting Standards (JARS) for quantitative research to enhance transparency and reproducibility [15].

### Inclusion and Exclusion Criteria

Judgments were included if they met the following criteria: (1) the presence of a diagnosed or alleged psychotic-spectrum disorder; (2) explicit reference to AI, technological surveillance, algorithmic systems, or related constructs within the delusional content; and (3) judicial discussion of dangerousness, detention status, conditional discharge, or treatment authorization. Both review board and civil authorization of care decisions were eligible where AI-themed beliefs were materially described in the reasoning. Cases were excluded if AI references were incidental, metaphorical, or unrelated to psychopathology (eg, references to AI tools used in evidence review). Judgments lacking sufficient factual detail to assess the relationship between AI-themed delusions and behavioral manifestations were also excluded.

### Participant (Case) Characteristics

The unit of analysis was the individual judicial decision rather than the accused, as multiple decisions concerning the same individual at different procedural stages were treated as distinct evaluative moments. As such, participant-level demographic variables (eg, age, sex, ethnicity, socioeconomic status) were inconsistently reported across judgments and could not be systematically analyzed. When available, these characteristics were extracted descriptively but were not used as inclusion criteria or covariates.

Multiple judgments pertaining to the same individual at different time points were to be treated as independent observations, reflecting distinct clinical and legal decision-making contexts. However, such judgments were not identified in this study.

### Sampling Procedures

Search terms included both French and English expressions (eg, “artificial intelligence,” “AI,” “psychosis,” “delusions,” “dangerosity,” “Tribunal,” “Review Board,” “temporary confinement,” and “treatment order”). Searches were conducted without date restrictions to capture the full emergence of AI-themed delusions in reported case law. Identified judgments were screened for relevance, and duplicate entries were removed. The final dataset consisted of decisions in which AI or related technological constructs were explicitly referenced in the factual findings, psychiatric evidence, or judicial reasoning. The full dataset is provided in Multimedia Appendix 1, with relevant judgment IDs from the SOQUIJ’s legal database. The full search strategy that was applied to identify judgments and construct the dataset is presented in Multimedia Appendix 2.

### Sample Size, Power, and Precision

The intended sample size was not predefined, as the study aimed to identify all eligible cases within the database. The achieved sample consisted of 29 judgments meeting the inclusion criteria. Given the exploratory nature of the study and the rarity of AI-themed delusions in published jurisprudence, no formal a priori power calculation was conducted. Analyses emphasized estimation (odds ratios [ORs] and 95% CIs) rather than statistical significance testing.

### Measures and Covariates

The primary independent variable was AI centrality within the delusional system, operationalized as a categorical variable (central vs noncentral) based on whether AI constituted the organizing framework of the delusional narrative.

AI centrality was operationalized as the degree to which AI functioned as the primary organizing principle of the delusional system. A case was coded as “central” when AI structured core explanatory mechanisms, including agency attribution (eg, AI controlling thoughts or actions), causal interpretation of events, or behavioral justification. In contrast, cases were coded as “noncentral” when AI appeared as a peripheral or incidental element within a broader delusional framework without organizing function. For example, cases were classified as AI-central when individuals described AI as directly controlling their behavior, issuing commands, or governing reality (eg, “The AI is directing my actions” or “The system controls what happens to me”). In contrast, cases were classified as AI-noncentral when AI was referenced without structural influence (eg, “AI is monitoring me” within a broader persecutory system not organized around AI).

The primary outcome was documented violence toward others, defined as any heteroaggressive behavior described in the judicial record.

Secondary outcomes included direct AI-linked violence attribution and judicial findings of significant public safety risk.

Covariates included lack of insight, treatment adherence, substance use disorder, and prior violence history, as described in tribunal reasoning.

### Data Collection

A structured coding grid was developed prior to data extraction to ensure standardized variable capture. Variables included demographic indicators (when available), psychiatric diagnoses, substance use history, prior violence, insight and treatment adherence, description of delusional themes, centrality of AI within the delusional system, explicit linkage between AI-themed beliefs and the index behavior, tribunal findings regarding significant public safety risk, and disposition outcomes (eg, detention, conditional discharge, authorization of care). AI centrality was coded categorically as “central,” “marginal,” “secondary,” or “not discussed” based on whether AI constituted the organizing framework of the delusional belief system or merely one thematic element among others. Violence variables were coded as “present” or “absent” for self-directed and other-directed behaviors, and, where possible, classified by severity.

### Quality of Measurements

Data extraction was conducted by authors AH and CP through full-text review of each judgment, including factual summaries, psychiatric expert testimony, and tribunal reasoning. To enhance reliability, ambiguous cases were reviewed twice, and coding decisions were linked to explicit textual citations from the judgment.

### Instrumentation

No standardized psychometric instruments were used in this study. All variables were derived from structured coding of judicial texts using an ad hoc coding framework developed for this study.

### Masking

Masking was not applicable, as data were derived from publicly available judicial records and no experimental manipulation or condition assignment was performed.

### Psychometrics

Formal psychometric validation of the coding framework was not conducted. However, the framework was designed to maximize construct clarity and reproducibility through explicit operational definitions and consensus-based coding procedures. Interrater reliability statistics were not calculated, given the exploratory nature of the study and the small sample size.

### Data Diagnostics

No cases were excluded after data extraction. Missing data were inherent to the use of judicial records and were coded as “not documented” when variables were not explicitly reported. No imputation procedures were applied. Given the small sample size, formal outlier detection and distributional transformations were not performed.

### Analysis

Descriptive statistics were first computed to characterize the distribution of AI centrality, violent behaviors, treatment adherence, and judicial outcomes within the dataset. The primary analytic objective was to examine the association between AI centrality and actual violence or judicial findings of significant public safety risk. AI centrality was dichotomized (central vs noncentral) for inferential testing. Bivariate associations between AI centrality and violence outcomes were assessed using Fisher’s exact test due to the small sample size.

ORs (95% CIs) were calculated to estimate the effect magnitude. Secondary analyses examined whether AI-themed delusions are explicitly linked to the behavior underlying the legal proceeding, distinguishing phenomenological presence from behavioral causation. Exploratory logistic regression models were constructed to evaluate whether AI centrality independently predicts violence after adjusting for established risk factors, such as lack of insight, treatment nonadherence, substance use, and prior violence history. Given the modest sample size, regression results were interpreted cautiously, emphasizing effect size and CIs rather than statistical significance alone. Qualitative thematic synthesis was additionally performed to contextualize quantitative findings, particularly regarding how tribunals conceptualized AI-themed delusions in relation to risk, insight, and treatment compliance.

### Ethical Considerations

As the data consisted exclusively of secondary analysis of publicly accessible, anonymized legal records, the study did not involve direct interaction with human participants and did not meet the definition of human subject research under applicable institutional and national guidelines. Accordingly, formal research ethics board review was not required.

No informed consent was obtained, as the study did not involve primary data collection. The original data (judicial decisions) are part of the public legal record, and their use for research purposes is permitted within the framework of open access legal information systems. All data used in this study were derived from deidentified judicial texts. No attempt was made to reidentify individuals, and no personally identifiable information beyond what is already publicly available in legal decisions was extracted or reported. In presenting results, care was taken to avoid inclusion of unnecessary identifying details, and all findings are reported in aggregate form.

No participants were recruited, and no compensation was provided, as this study did not involve human subjects. No images, figures, or supplementary materials include identifiable individuals. All content is derived from textual legal sources, and no individual-level identification is possible from the data presented.

## Results

### Sample Characteristics and Flow

A total of 29 judgments met the inclusion criteria and were retained for analysis. In all cases, AI was explicitly referenced within the delusional content described in the judicial record. AI centrality within the psychotic framework was coded dichotomously as central (including cases coded as “secondary to central”) or noncentral (including cases coded as “marginal,” “secondary,” or “not discussed”). AI was considered central in 15 (51.7%) cases and noncentral in 14 (48.3%) cases.

Across the sample, dangerosity toward others, as determined in tribunal reasoning or based on documented heteroaggressive behavior, was identified in 20 (69%) cases (95% CI 50.3-83.1), whereas 9 (31%) cases did not involve a finding of violence toward others. Thus, the overall base rate of violence toward others in this forensic sample was high. Characteristics of the judgments are found in Table 1.

**Table 1:** Descriptive characteristics of forensic psychiatric judgments (N=29) involving AI^a^-related delusions.

Characteristics			Judgments, n (%)
**AI centrality**			
	Central (including secondary to central)	15 (51.7)	
	Noncentral (marginal, secondary, not discussed)	14 (48.3)	
**Violence toward others^b^**			
	Present	20 (69.0)	
	Absent	9 (31.0)	
**Direct AI-** **linked** **violence attribution**			
	Direct causal attribution	11 (37.9)	
	Indirect or none	18 (62.1)	
**Judicial findings of significant public safety risk^c^**			
	Risk confirmed by tribunal	22 (75.9)	
	No significant risk found	7 (24.1)	
**Judicial disposition**			
	Continued detention/maintenance of custody	15 (51.7)	
	Conditional discharge (with modalities)	10 (34.5)	
	Absolute discharge	4 (13.8)	
**Documented lack of insight**			
	Present (partial or absent insight)	23 (79.3)	
	Adequate insight	6 (20.7)	
**Treatment refusal or nonadherence**			
	Present	17 (58.6)	
	Absent/adherent	12 (41.4)	
**Substance use disorder documented**			
	Present	14 (48.3)	
	Not documented	15 (51.7)	

^a^AI: artificial intelligence.

^b^Violence toward others refers to documented heteroaggressive behavior described in the judicial record.

^c^Judicial findings of significant public safety risk reflect tribunal determinations of dangerousness.

### AI Centrality and Violence Toward Others

As shown in Table 2, cases in which AI was coded as a central component of the delusional or explanatory framework were more likely to involve documented violence toward others compared to cases in which AI was marginal or absent. Specifically, violence toward others was observed in 80.0% (12/15) of AI-central cases (95% CI 54.8-93.0) versus 57.1% (8/14) of AI-noncentral cases (95% CI 32.6-78.6). This corresponded to an unadjusted OR of 2.91 (95% CI 0.63-13.45). Although the magnitude of the association suggests a potentially meaningful increase in the likelihood of violence when AI content is central, the difference did not reach statistical significance using Fisher’s exact test (*P*=.26).

**Table 2:** AI^a^ centrality and violence toward others.

Predictor and outcomes		AI-central/exposed, n/N (%); 95% CI	AI-noncentral/unexposed, n/N (%); 95% CI	Effect size, OR^b^ (95% CI)	*P* value (Fisher’s exact test)
**AI centrality^c^**					
	Violence toward others^d^	12/15 (80.0); 54.8-93.0	8/14 (57.1); 32.6-78.6	2.91 (0.63-13.45)	.26
	Direct AI-linked violence attribution^e^	9/15 (60.0); 35.8-80.2	2/14 (14.3); 4.0-39.9	9.00 (1.48-54.6)	.01
	Judicial findings of significant public safety risk^f^	13/15 (86.7); 62.1-96.3	9/14 (64.3); 38.8-83.7	3.60 (0.63-20.5)	.24
**Direct AI-linked violence attribution**					
	Violence toward others	10/11 (90.9); 62.3-98.4	10/18 (55.6); 33.7-75.4	8.00 (0.87-73.7)	.07
	Judicial findings of significant public safety risk	10/11 (90.9); 62.3-98.4	12/18 (66.7); 43.7-83.7	4.00 (0.39-41.3)	.22

^a^AI: artificial intelligence.

^b^OR: odds ratio.

^c^AI centrality was dichotomized as central versus noncentral based on structured coding of delusional system organization.

^d^Violence toward others was defined as documented heteroaggressive behavior.

^e^Direct AI-linked violence attribution refers to the explicit causal linkage between AI-related beliefs and the index behavior.

^f^Judicial findings of significant public safety risk reflect tribunal determinations of dangerousness.

Importantly, although AI centrality was strongly associated with explicit AI-linked violence attribution (OR 9.00, 95% CI 1.48-54.6; *P*=.01), its relationship with actual violent behavior was more moderate and statistically nonsignificant. This pattern suggests that AI centrality may be more associated with the cognitive framing and narrative organization of violence rather than serving as an independent predictor of violent conduct per se. However, the wide CIs reflect substantial uncertainty around the effect estimate, consistent with limited statistical power in this sample (N=29), and the data remain compatible with both increased and decreased risk.

These findings indicate that within this dataset, centrality of AI-themed delusions was not statistically associated with increased violence toward others.

### Qualitative Pattern of Judicial Reasoning

A detailed review of tribunal reasoning further contextualizes this statistical pattern. In cases where violence was affirmed, the justification for detention or restrictive measures rarely emphasized AI content as a primary causal factor. Instead, judicial decisions consistently highlighted persistent psychotic symptoms, impaired insight, treatment nonadherence, substance use, prior violent behavior, impulsivity, and instability of living conditions.

AI-related delusional material was typically described as part of the phenomenological expression of the psychosis, often embedded within persecutory, grandiose, or referential themes. Even in cases where AI content was central and ontologically elaborate, tribunals tended to ground their assessment of risk in behavioral history and clinical instability rather than in the thematic content of the delusion itself.

In contrast, several cases in which AI was central but violence was absent were characterized by improved insight, medication adherence, therapeutic alliance, and stable supervision. In those instances, tribunals explicitly noted clinical stabilization and controlled risk under structured conditions. The thematic typology of AI-related delusions is found in Table 3.

**Table 3:** Thematic typology of AI^a^-related delusions in forensic psychiatric judgments.

Thematic category^b^	Operational definition	Core delusional structure	Cases (N=29), n (%)	AI-central cases^c^ (n=15), n (%)	AI-noncentral cases^c^ (n=14), n (%)
AI as a controlling agent	AI described as externally directing thoughts, behavior, or life events	Externalized agency; behavioral control; algorithmic directives	12 (41.4)	10 (66.7)	2 (14.3)
AI as a persecutory system	AI portrayed as surveilling, torturing, targeting, or harming the individual	Technological paranoia; surveillance; implanted devices	14 (48.3)	11 (73.3)	3 (21.4)
AI as a command source	AI issuing explicit instructions or moral imperatives	Command structure; authority-based obedience	6 (20.7)	5 (33.3)	1 (7.1)
AI as a grandiose creation	Individual claims to have created, mastered, or uniquely understood AI	Narcissistic expansion; technological omnipotence	8 (27.6)	6 (40.0)	2 (14.3)
AI as an ontological reality framework	AI used to reframe the nature of existence (simulation, world creation, universal system)	Cosmological restructuring; reality substitution	7 (24.1)	6 (40.0)	1 (7.1)
AI as a moral/existential justification	AI used to justify actions, destiny, or mission	Externalized moral authority	9 (31.0)	7 (46.7)	2 (14.3)
AI as a marginal referential element	AI referenced but not structurally central to the delusional system	Peripheral thematic content	14 (48.3)	0	14 (100.0)

^a^AI: artificial intelligence.

^b^Categories represent recurring patterns of AI-related delusional content identified through qualitative thematic synthesis.

^c^AI-central cases (n=15, 51.7%) refer to judgments in which AI functioned as the primary organizing structure of the delusional system, whereas AI-noncentral cases (n=14, 48.3%) refer to peripheral or incidental AI content.

### Interpretation of the Association

Table 4 presents the distribution of core forensic risk factors according to AI delusional centrality. When Tables 2 and 4 are considered together, a coherent pattern emerges: AI centrality is not independently associated with violent behavior toward others, but it appears to cluster with markers of epistemic vulnerability that are themselves relevant to forensic risk formulation. In Table 2, the proportion of cases involving violence toward others did not differ significantly between AI-central and AI-noncentral cases, and effect sizes were modest with wide CIs crossing unity. This indicates that AI occupying a structurally central position within the delusional system does not, in this sample, translate into a statistically detectable increase in heteroaggressive conduct.

**Table 4:** Distribution of core forensic risk factors according to AI^a^ delusional centrality^b^.

Risk variable	AI-central cases (n=15), n (%)	AI-noncentral cases (n=14), n (%)	OR^c^ (95% CI)^d^	*P* value
Lack of insight	13 (86.7)	8 (57.1)	4.89 (0.79-30.1)	.09
Refusal of treatment	9 (60.0)	4 (28.6)	3.75 (0.74-18.9)	.14
Substance use disorder	10 (66.7)	7 (50.0)	2.00 (0.42-9.46)	.46
Prior violence history	8 (53.3)	6 (42.9)	1.52 (0.33-7.05)	.72
Judicial findings of significant public safety risk	11 (73.3)	7 (50.0)	2.75 (0.57-13.3)	.27

^a^AI: artificial intelligence.

^b^AI centrality was defined based on whether AI constituted the organizing framework of the delusional system.

^c^OR: odds ratio.

^d^ORs (95% CIs) indicate between-group differences.

However, Table 4 refines this interpretation by demonstrating that AI-central cases exhibit a stronger concentration of cognitive and clinical risk indicators. The most pronounced association concerns lack of insight, which was markedly more prevalent in AI-central cases, with an OR approaching 5, albeit with a wide CI, reflecting limited statistical power. Treatment refusal also trended higher in the AI-central group, with an OR suggestive of a clinically meaningful effect size, though again not reaching statistical significance. In contrast, classical criminogenic variables, such as prior violence history and substance use disorder, were only weakly associated with AI centrality, with effect estimates close to unity and nonsignificant *P* values. Furthermore, the model demonstrated minimal explanatory value (pseudo R²=0.001), and CIs were wide, reflecting substantial imprecision. These findings should therefore be interpreted as inconclusive.

This pattern suggests that AI centrality may function less as a direct behavioral risk amplifier and more as a structural indicator of delusional system consolidation. In other words, when AI is positioned as a primary agent within the individual’s explanatory framework (controlling events, issuing commands, or structuring reality), the psychotic architecture appears more entrenched and less permeable to corrective feedback. This interpretation is consistent with the elevated prevalence of insight impairment and treatment nonadherence observed in Table 4, while remaining aligned with the absence of a statistically significant increase in the overt violence observed in Table 2.

Judicial findings of significant public safety risk were more frequent in AI-central cases, but this association did not reach statistical significance and displayed a moderate effect size with wide CIs. This may indicate that tribunals are responding, not to documented increases in violent behavior per se, but to the overall clinical presentation characterized by impaired insight, perceived instability, and risk of decompensation. Thus, the association between AI centrality and judicial outcomes appears mediated by broader clinical risk constructs rather than by direct violence linkage.

These findings support a more nuanced interpretation: AI centrality is associated with epistemic disturbance and vulnerability within the psychotic system, but it does not independently predict violence toward others in this cohort. The clinical and forensic relevance of AI centrality may therefore lie in its relationship to insight erosion and treatment instability rather than in direct behavioral escalation.

### Implications for Forensic Psychiatry

From a forensic psychiatry perspective, these results challenge potential assumptions that technologically themed delusions represent a uniquely destabilizing or violence-enhancing subtype of psychosis. Instead, AI-themed delusions appear to mirror historical shifts in persecutory or grandiose content, reflecting sociocultural embedding of psychotic material without fundamentally altering the behavioral risk architecture.

In this sample of Quebec forensic judgments, tribunals implicitly recognized this distinction, focusing decisively on clinical stability and behavioral history rather than on the thematic novelty of AI-related beliefs. The data therefore support the interpretation that AI centrality is phenomenologically salient but not independently predictive of violence.

**Figure 1:**
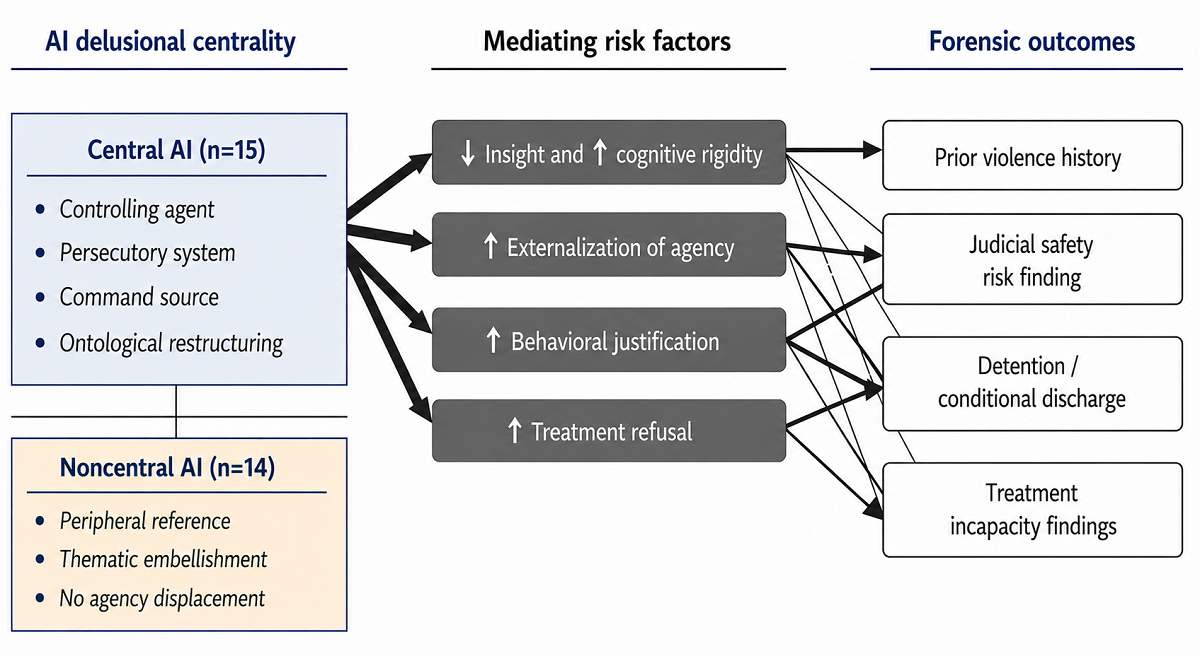
Structural model of AI delusional centrality and forensic risk pathways. The model illustrates how AI centrality within delusional systems (defined as AI functioning as a controlling agent, persecutory system, command source, or ontological framework) is hypothesized to influence forensic outcomes indirectly through mediating mechanisms, including impaired insight, cognitive rigidity, externalization of agency, and treatment nonadherence. These intermediate factors are associated with downstream outcomes, such as violence toward others, judicial findings of significant public safety risk, and restrictive dispositions. The model reflects observed empirical patterns and theoretical integration rather than causal inference. AI: artificial intelligence.

Figure 1 illustrates the proposed structural model linking AI delusional centrality to forensic risk outcomes. In this framework, AI-related content is not treated as a homogeneous thematic element but is differentiated according to its structural role within the delusional system. When AI occupies a central position (functioning as a controlling agent, persecutory system, command source, or ontological framework) it is hypothesized to reorganize agency attribution and meaning-making processes. This centrality appears to operate through mediating risk mechanisms, including diminished insight, increased cognitive rigidity, externalization of agency, behavioral justification grounded in delusional logic, and higher likelihood of treatment refusal. These mediators, in turn, are associated with downstream forensic outcomes, such as prior history of violence, judicial findings of significant public safety risk, detention or conditional discharge decisions, and determinations of treatment incapacity. In contrast, when AI content remains peripheral or decorative within the delusional narrative, it does not appear to exert the same structural influence on agency, treatment engagement, or judicial risk assessment. The model therefore conceptualizes AI delusional centrality as a qualitative intensifier of established criminogenic and clinical risk processes rather than as an isolated thematic variable.

## Discussion

### Principal Findings

This jurisprudential analysis of 29 Quebec forensic judgments found that AI-related beliefs were frequently embedded within persecutory and agency-displacing psychotic narratives and that the overall base rate of heteroaggressive behavior or violence toward others was high in this tribunal-derived sample. Across decisions, tribunals most consistently anchored their reasoning in conventional clinical and behavioral determinants of risk (impaired insight, treatment engagement, persistence of psychotic symptoms, substance use, and prior violence), while AI content was often treated as a phenomenological expression of illness rather than a stand-alone driver of dangerousness.

The central finding was that AI centrality is not statistically significantly associated with the occurrence of violence; however, effect estimates were imprecise and the study was underpowered, limiting the ability to draw definitive conclusions regarding the presence or absence of an association. In contrast, AI centrality was strongly associated with explicit AI-linked violence attribution, indicating that when AI is structurally central to the delusional architecture, violence is more often framed as being caused, commanded, justified, or organized by the AI narrative. Stratified patterns further suggested that AI centrality tends to co-occur with markers of epistemic vulnerability (especially impaired insight and treatment refusal) more than with classical criminogenic variables, such as prior violence history or substance use disorder. AI centrality is therefore suggested to function as a structural organizer of agency and meaning-making that may influence judicial risk reasoning indirectly through insight and adherence mechanisms rather than as a direct behavioral risk amplifier.

### Interpretation and Comparison With Prior Work

The question of whether AI-themed delusions represent a qualitatively new risk construct must be situated within the broader phenomenology of persecutory and passivity delusions. Empirical work on threat anticipation and persecutory ideation demonstrates that the structure of paranoid belief formation is typically driven by cognitive-affective processes rather than by specific thematic content [16,17]. Freeman et al [16] showed that persecutory delusions are maintained through misinterpretations of social threat and safety behaviors that prevent disconfirmation. Similarly, research on anomalous experiences and belief evaluation indicates that delusional conviction is shaped by reasoning biases and affective dysregulation rather than the cultural substrate of the belief [17]. Within this framework, AI can be understood as a contemporary narrative vehicle for long-standing mechanisms of agency externalization and threat attribution. The findings of this study, demonstrating that AI centrality does not independently predict violence, align with this cognitive model: the thematic novelty of AI does not appear to supersede the underlying psychological architecture of paranoia.

The distinction between thematic content and structural role is also conceptually important. Delusional themes (eg, surveillance, control, persecution) often reflect the sociocultural context, whereas structural features refer to how beliefs organize agency, causality, and behavioral meaning within the psychotic system. AI was conceptualized as structurally central when it functioned as a primary agent within this architecture, shaping not only the content of beliefs but also the explanatory logic linking perception, intention, and action. This distinction is grounded in cognitive models of psychosis, which emphasize that behavioral consequences are more strongly determined by belief organization, conviction, and integration than by thematic content alone [16,17]. From this perspective, AI centrality is therefore not only a thematic variation but also a potential marker of system-level consolidation of delusional belief.

From a violence risk perspective, the results are consistent with longitudinal evidence demonstrating that psychotic symptoms alone are weak predictors of violent outcomes when separated from dynamic contextual variables. Large epidemiological and register-based studies [18,19] have indicated that violence risk in psychotic disorders is substantially moderated by substance misuse, prior violence, and social instability rather than by specific delusional themes. Swanson et al [18] showed that comorbid substance use and criminogenic history account for much of the excess violence risk observed in schizophrenia-spectrum disorders. Fazel et al’s [19] population-based investigations further demonstrated that familial and environmental confounders attenuate the association between psychosis and violence, underscoring the primacy of broader risk ecology. Moreover, research examining “threat/control-override” symptoms suggests that only specific configurations of persecutory beliefs combined with affective arousal predict violence, and even then, effect sizes are modest [20]. This study’s pattern (where AI centrality clustered more strongly with impaired insight and treatment refusal than with prior violence) therefore supports the interpretation that AI operates as a structural intensifier of delusional organization rather than as an independent criminogenic variable.

Judicial decision-making research further contextualizes these findings. Studies of forensic risk communication demonstrate that tribunals often rely on narrative coherence and perceived treatability when translating psychiatric evidence into legal determinations [21,22]. Large-scale analyses of insanity acquittees and review board populations indicate that SPJ and clinical stability markers dominate disposition decisions, even when symptom content is vivid or unusual [23]. Research on stigma and perceived dangerousness also suggests that technologically mediated or “modern” delusional themes may amplify perceived unpredictability, yet actual risk judgments remain anchored to behavioral history and compliance indicators [24]. In this light, the findings of this study (showing strong associations between AI centrality and AI-linked violence attribution but not with actual violence) suggest that AI may influence the explanatory framing of behavior without fundamentally altering tribunals’ reliance on established risk variables. This distinction between narrative attribution and behavioral prediction is consistent with prior forensic scholarship emphasizing the need to disentangle phenomenology from empirically supported risk mechanisms.

### Implications for Clinical and Forensic Practice

Clinically, these findings reinforce the importance of assessing delusional structure rather than thematic novelty. When AI occupies a central role (functioning as a controlling agent, persecutory system, or moral authority), clinicians are encouraged to evaluate associated markers of epistemic rigidity, diminished insight, and impaired treatment engagement. Evidence-based cognitive models of paranoia emphasize collaborative belief examination, reduction in safety behaviors, and enhancement of metacognitive awareness as key interventions [16,17]. Given the well-established association between nonadherence and adverse outcomes, including violence in high-risk populations [18,20,25], management strategies in AI-central cases should prioritize alliance building, adherence monitoring, and substance use stabilization rather than focusing exclusively on the technological content of the delusion. Inquiry into AI use should be routine, framed neutrally, and integrated into broader assessment of digital behavior patterns that may reinforce maladaptive explanatory systems.

For forensic tribunals, the implications are conceptual and procedural. AI-themed delusions may appear uniquely alarming because they resonate with real-world technological anxieties, but empirical evidence continues to support reliance on dynamic risk factors (clinical stability, substance use, behavioral history, and adherence capacity) as the strongest predictors of future harm [19,23,26]. SPJ approaches remain appropriate, provided they are grounded in individualized formulation rather than narrative salience [22]. Explicit differentiation between the plausibility of certain technological fears in contemporary society and the individual’s demonstrated behavioral risk profile may reduce novelty bias and promote proportionate dispositions. As digital technologies become increasingly embedded in everyday life, forensic systems will require refined assessment frameworks that address technologically mediated delusions without conflating cultural modernization with categorical dangerousness.

### Limitations

Several limitations qualify interpretation. First, the sample size was modest (N=29), producing wide CIs and limited power to detect anything but large effects; null findings regarding violence and judicial risk should therefore be interpreted as inconclusive rather than definitive absence of association. Second, the dataset was constructed from publicly available judgments, which are not designed for research purposes and reflect selection processes in publication and documentation. Decisions that are written, reported, and indexed may differ systematically from those that are not, and the level of clinical detail varies by tribunal, legal issue, and authoring member, increasing the risk of information bias. Third, the unit of analysis was the judgment rather than the individual; where multiple decisions concern the same person across time, observations may not be independent, and temporal dynamics of symptoms and risk management may be conflated with cross-sectional comparisons. Fourth, key variables were coded from judicial descriptions of clinical evidence; misclassification is possible where judgments summarize testimony selectively or omit negative findings. Fifth, AI centrality is a constructed exposure variable requiring interpretive coding and remains vulnerable to coder subjectivity and differential documentation. Finally, generalizability is limited, as Quebec review board processes and reporting practices differ from other jurisdictions and the sample likely overrepresents severe and unstable cases.

### Conclusion

In conclusion, this study demonstrated that AI-related delusional content is no longer a peripheral narrative feature in forensic psychiatric judgments, but in a substantial proportion of cases, it functions as a structurally central element of the psychotic system. When AI themes were central rather than marginal, they were more frequently associated with persistent lack of insight, treatment refusal, substance use comorbidity, prior violence, and formal judicial findings of significant public safety risk. These findings suggest that AI delusions may operate, not as contemporary cultural symbols, but as organizing frameworks that shape risk appraisal, perceived persecution, and behavioral dyscontrol. At the same time, centrality did not automatically determine outcome; clinical stabilization, adherence, and structured supervision remained decisive moderators of risk. By integrating it with forensic risk variables, this work provides an empirically grounded typology of AI delusional centrality within real-world judicial decision-making. The results highlight the need for nuanced psychiatric assessments that differentiate thematic novelty from structural pathogenicity. As digital technologies continue to permeate everyday life, courts and clinicians will increasingly confront technologically mediated delusional systems. Future research should examine longitudinal trajectories, treatment responsiveness, and cross-jurisdictional patterns to better inform forensic practice and public safety policy in the era of digital psychiatry.

## Appendix

Multimedia Appendix 1 Full dataset that was used for this study.

Multimedia Appendix 2 Search strategies to identify the judgments.

## Abbreviations

AI: artificial intelligence
HCR-20: Historical Clinical Risk Management-20
NCRMD: not criminally responsible on account of mental disorder
OR: odds ratio
SOQUIJ: Société québécoise d’information juridique
SPJ: structured professional judgment
