# Supplementary material for: Artificial Intelligence Centrality in Psychotic Delusions and Violence Risk in Forensic Psychiatry: Retrospective Observational Study of Judicial Decisions
Source: J Med Internet Res. 2026 May 20;28:e93349. doi: 10.2196/93349 (PMC13234535; doi:10.2196/93349)
Supplement: Multimedia Appendix 2 [file jmir_v28i1e93349_app2.pdf]

Search terms and search strategies

1) Keyword Bank - SOQUIJ Search

Objective: Identify decisions in which artificial intelligence is integrated into delusional content, pathological beliefs, or reported symptomatology (rather than being mentioned in a neutral technological context).

A. Core Terms - Artificial Intelligence

French (translated into English equivalents)

artificial intelligence

AI

algorithm

intelligent machine

intelligent system

digital entity

autonomous technology

digital intelligence

intelligence of the future

English

artificial intelligence

AI

algorithm*

intelligent system*

machine intelligence

autonomous system*

digital intelligence

B. AI Integrated into Symptomatology (Typical Delusional Formulations)

French (translated into English equivalents)

controlled by artificial intelligence

influenced by artificial intelligence

directed / commanded by AI

messages sent by AI

voices originating from artificial intelligence

technological implants

internal technological devices

technological surveillance

algorithmic manipulation

artificial intelligence coming from the future

artificial intelligence dictating actions

technological delusion

technological control delusion

English

controlled by artificial intelligence

influenced by AI

commanded by AI

AI telling him/her to act

messages from AI

technological implants

technological control delusion

AI-driven delusions

persecutory technological delusions

future AI controlling behavior

2) Search strategies

For all courts on SOQUIJ : use all the above-mentioned keywords to identify the relevant judgements since their inception up to December 2025.
